# Inflammatory Bowel Disease and X (Formerly Twitter) Influencers: Who Are They and What Do They Say?

**DOI:** 10.7759/cureus.47536

**Published:** 2023-10-23

**Authors:** Ayushi Garg, Aalam Sohal, Shivam Kalra, Carol Singh, Ishandeep Singh, Jasneet Grewal, Rohin Kansal, Kashish Malhotra, Ramit Mahajan, Vandana Midha, Arshdeep Singh, Ajit Sood, Ashvind Bawa

**Affiliations:** 1 Internal Medicine, Dayanand Medical College and Hospital, Ludhiana, IND; 2 Internal Medicine, UCSF Fresno, Fresno, USA; 3 Internal Medicine, Trident Medical Center, North Charleston, USA; 4 General Surgery, Dayanand Medical College and Hospital, Ludhiana, IND; 5 Gastroenterology, Dayanand Medical College and Hospital, Ludhiana, IND

**Keywords:** public awareness, social media, influencers, inflammatory bowel disease (ibd), x (formerly twitter)

## Abstract

Background and objective

More than half of the population suffering from inflammatory bowel disease (IBD) use the internet as a primary source of information on their condition. X (formerly Twitter) has been increasingly used to disseminate healthcare-related information. In this study, we aimed to identify top influencers on the topic of IBD on X and correlate the relevance of their social media engagements with their professional expertise or academic productivity.

Methods

X (formerly Twitter) influence scores for the search topic IBD were obtained using Cronycle API, a proprietary software employing multiple algorithms to rank influencers. Data regarding gender, profession, location, and research productivity represented as h-index was collected.

Results

We collected information on the top 100 IBD influencers on X. The majority of influencers were gastroenterologists, followed by IBD advocates. Of note, 62% of the IBD influencers were from the US followed by the UK and Canada. A positive correlation was observed between the X topic score and the h-index of the influencer (r=+0.488, p<0.001)

Conclusions

The strong correlation observed between the X topic score and h-index suggests that social media is a viable platform for gaining information regarding IBD. Further research aimed at counteracting misleading information by providing facts and data in a succinct manner about IBD on social media is required to improve disease awareness.

## Introduction

Social media has been increasingly used by healthcare providers to disseminate information regarding various diseases. The coronavirus disease 2019 (COVID-19) pandemic has further shifted the dynamics of the IBD advocator-doctor relationship thanks to widespread outreach and ease of availability of social media [1,2]. While increased patient knowledge may facilitate the shared decision-making process, the accuracy and extent of information available on social media are frequently incoherent and unfiltered. Thus, unregulated healthcare-related communication may mislead patients into making poor choices, as well as opting for ineffective treatment and unproductive disease management [3]. While scientific and evidence-based publications are still the point of reference for academics, the rising visibility and significance of social media in providing healthcare-related information are factors that cannot be ignored and need to be evaluated. A physician’s ability to direct a patient’s online research toward reliable and authentic information will lead to improved outcomes and enhance the physician-patient relationship [2,4]. Dissemination of relevant information and providing avenues for gaining emotional support will facilitate the effective translation of correct medical knowledge into improved and safe healthcare practices [5].

The term “influencer” in the context of social media refers to people who have built a significant following on various social media platforms/sites and are known for their knowledge and proficiency in a particular field. Such "influencers" have a significant digital footprint and consumer engagement. Available statistics have illustrated the significance of an influencer in setting trends or driving thinking, with the influencer marketing sector valued at $16.4 billion [6,7]. Apart from the financial considerations, it is important to recognize that top medical professionals, such as doctors, nurse practitioners, physician assistants, and patients themselves donning the role of influencers and projecting themselves as strong voices in their particular domains can have a significant impact on improving healthcare [7,8,9]. The fact that social media platforms such as X (formerly Twitter) play a crucial role in terms of raising awareness and providing critical information about various chronic diseases such as inflammatory bowel disease (IBD) makes it imperative to draw attention to the pivotal role social media can play to influence decision-making about its course and treatment options for improved outcomes. Its multifactorial etiology constituted by Crohn's disease and ulcerative colitis can contribute significantly to morbidity or mortality among patients [10,11]. The chronic course of IBD significantly hampers the patient’s quality of life, making it very important to cultivate an effective relationship between patient and their physicians to increase IBD advocator satisfaction and achieve better outcomes [12].

Nevertheless, significant disparities in the patient-physician ratio, time constraints limiting in-depth inquiry, and lack of consistent emotional support warrant the creation of a space where people can communicate effectively and fruitfully. Furthermore, the stigmatization associated with IBD, at par or even higher than that with HIV/AIDS or diabetes, and the sense of embarrassment due to the disruption of normal activities drive most patients with IBD toward social media to obtain peer support and disease-related information and to communicate with their physicians or IBD support-related organizations [13,14]. Despite the presence of several online platforms, the easy accessibility and succinct information provided by X make it a noteworthy medium for providing a symbiotic, direct, and bidirectional flow of health-promoting information that transcends the walls of the clinic [15,16]. Furthermore, this interdependent procurement of information enables physicians to be up-to-date about trends in contemporary medicine. Therefore, with a view to analyzing the active engagement of IBD patients and healthcare providers via the X platform, the present study aims to assess the role and impact of key stakeholders or prominent influencers who with their every tweet raise awareness about IBD.

Though these X influencers boast tremendous popularity with their active social media participation and are associated with massive outreach, their credibility in providing authentic information related to IBD remains a matter of crucial concern. For instance, the evaluation of IBD-related content on YouTube by Mukewar et al. [17] revealed that the popularity of these influencers often belied the poor quality of their content and the lack of depth and accuracy. Thus, identifying individual top IBD influencers on X will aid in shedding light on the challenges and advantages of having a readily available platform to disseminate credible IBD-related education beyond the realms of clinics and hospitals.

## Materials and methods

Data were obtained using the Cronycle API on March 31, 2022, in order to identify X influencer scores for the search topic IBD. Cronycle is a software that helps to identify emerging trends and insights and helps filter data containing specific keywords. It quantifies influencer scores based on the number of connections (followers/following) and engagements (views, likes, and retweets). It employs graph partitioning techniques to determine influencer scores relevant to specific topic searches, allowing the topmost influential X (formerly Twitter) accounts to be ranked. The credibility of this proprietary software in assessing top influencers has been established in previous studies in the fields of gastroenterology [18], critical care medicine [19], and colorectal surgery [20]. We excluded organizational accounts from this study.

The accounts with top influencer scores were connected to individual names, and accounts were classified with regard to the influencer’s profession, practice setting, location, and h-index. Public resources were searched, including LinkedIn and other institutional or practice websites, to collect this information. The Spearman correlation coefficient was used to identify the relationship between X influencer scores and h-index. A p-value <0.05 was considered statistically significant for this study. Graphical and statistical analysis was performed using Microsoft Excel and the open-access JASP (Jeffreys’s Amazing Statistics Program).

## Results

**Figure 1:**
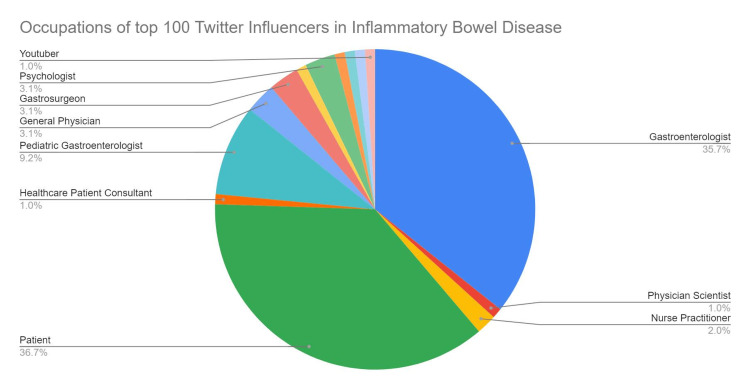
Breakdown of the professional backgrounds of the top 100 Twitter influencers in inflammatory bowel disease

We collected data on the 100 most popular influencers tweeting about IBD. There were 54% (54) males and 46% (46) females in the group. The majority of the individuals with the top Twitter scores were IBD advocates (37%, 37 influencers), followed by adult gastroenterologists (36%, 36 influencers) and pediatric gastroenterologists (9%, nine influencers). The other tweeters were gastroenterology surgeons, physicians, psychologists, nurse practitioners, and healthcare consultants (Figure 1).

Data regarding X handles, profession, topic score, and h-index are presented in Table 1.

**Table 1:** Profession/role with the sum of h-index The data has been represented as N

Profession/role	Sum of h-index
Broadcaster	0
Gastroenterologist	830
Gastrosurgeon	78
General physician	66
Healthcare patient consultant	0
IBD researcher+patient	0
Nurse practitioner	0
Patient	13
Patient advocate	0
Patient advocate/medical laboratory scientist	0
Patient+journalist	0
Patient+nutritionist	0
Pediatric gastroenterologist	98
Physician-scientist	60
Psychologist	49
Writer	0
Youtuber	0
Grand total	1194

**Figure 2:**
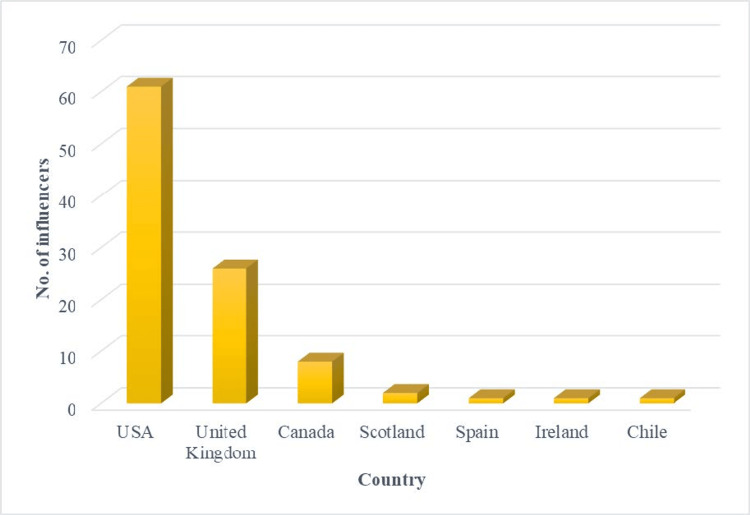
Distribution of the top 100 Twitter influencers in inflammatory bowel disease by country

Our study noted that the majority of influencers tweeting about IBD were from the US (61%, 61 influencers), followed by the UK (26%, 26 influencers), Canada (8%, eight influencers), Scotland (2%, two influencers), Ireland (1%, one influencer), Spain (1%, one influencer), and Chile (1%, one influencer), as shown in Figure 2.

**Figure 3:**
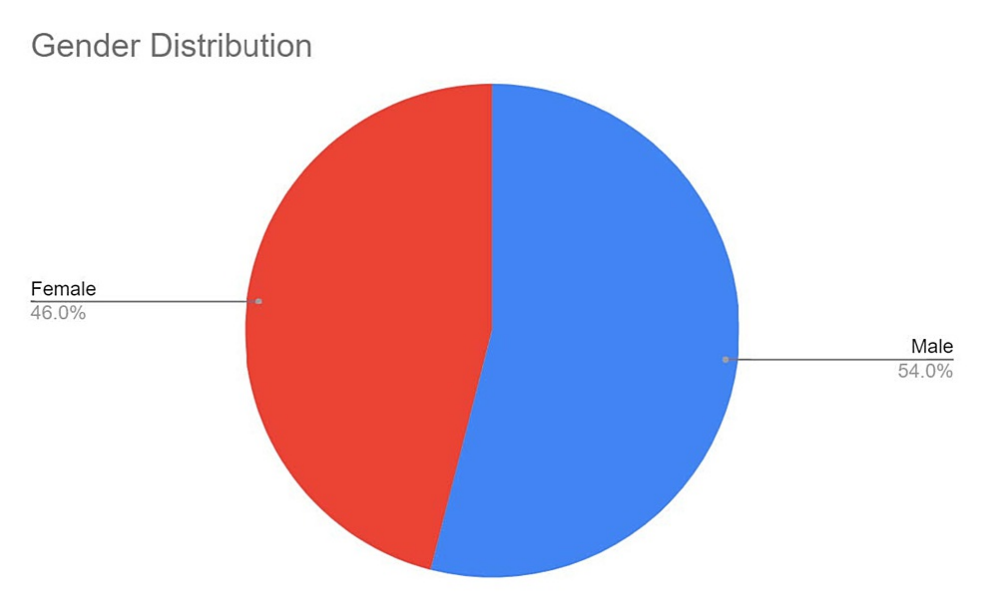
Graphical presentation of gender distribution

On examining the academic productivity, the data exhibited skewness due to a wide array of outliers, but the h-index varied from 1 to 149 with a median of 17. Nevertheless, the gender disparity in research was evident in the results obtained as the male population had a higher h-index as compared to the female population, as shown in Figure 3.

**Figure 4:**
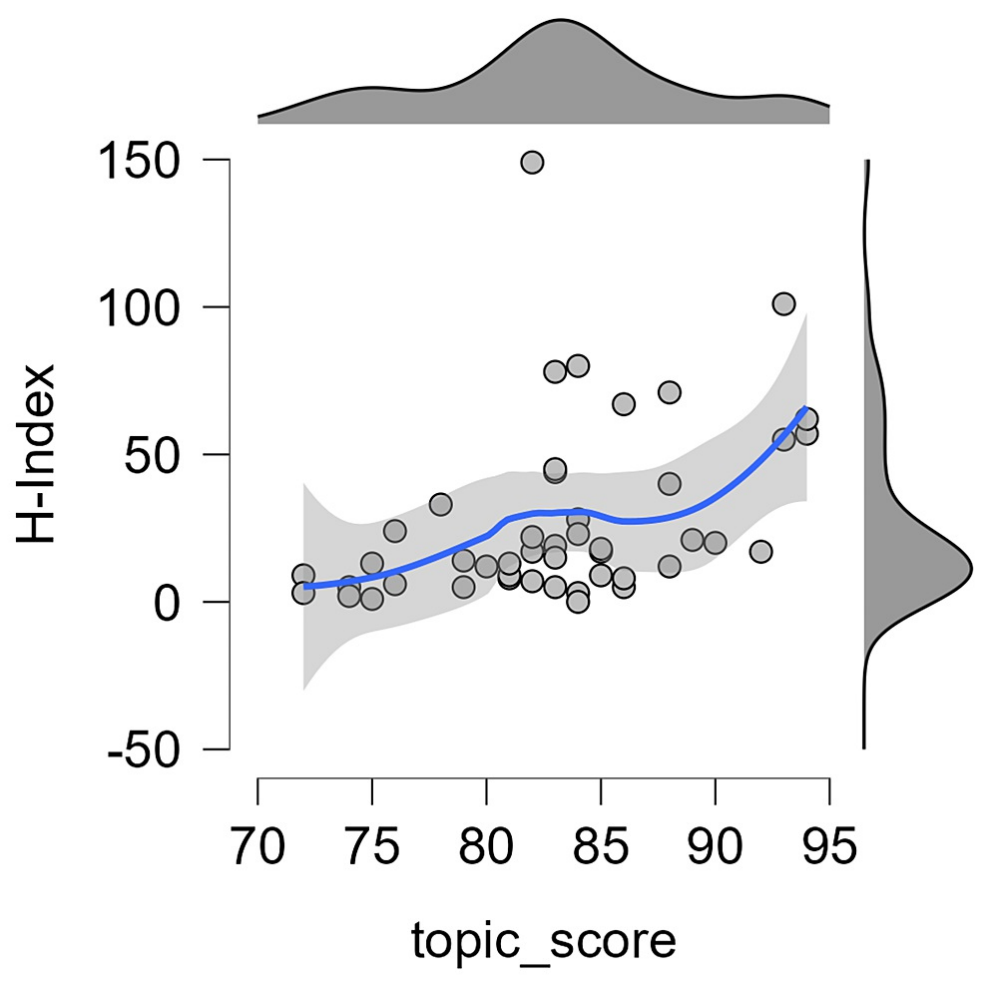
Correlation between the topic score and h-index The data has been represented as N and the p-value is statistically significant (p<0.001)

A statistically significant (p<0.001) moderately positive correlation was observed between the topic score and h-index with a Spearman correlation coefficient of +0.488 (Figure 4).

## Discussion

Based on our findings, the information provided by the top X influencers in IBD is in concordance with their academic expertise. Social media has made revolutionary progress over the last decade and has contributed significantly to the medical profession with regard to the effortless exchange of information among healthcare professionals and the general population. It is pertinent that the information provided via digital mediums is accurate; our present work in investigating the influencers leading the discussion about IBD on X found active participation by gastroenterologists (Figure 1). There has been a growing social media presence of various gastrointestinal societies such as the American Gastroenterological Association (AGA), American College of Gastroenterology (ACG), and American Association for the Study of Liver Diseases (AASLD), which points to the critical role of X as a powerful platform for swiftly and effectively sharing medical knowledge, connecting with colleagues, and expanding one's professional network. A study analyzing tweets of 16 IBD specialists from prestigious medical colleges in the US revealed that 53% of the tweets pertained to research sharing among peers [16].

There is a need to expand the outreach and improve patient engagement by filling knowledge gaps with easily comprehensible and digestible authentic information that will also aid in disseminating research and increase the reach of one's practice or institution while advocating for the advancement of healthcare. Our study noted that the majority of the top 100 X influencers were from developed nations, i.e., the United States, United Kingdom, and Canada were the forerunners leading the tribe of IBD influencers, which could be attributed to better access to infrastructural facilities, high usage and outreach of information technology, as well as more sensitized policies aligned towards improving healthcare in these countries. The same trend of developed nations being the prime participants has been observed with regard to influencers leading the discussion in various other specialties such as cardiology [21], neurosurgery [22], orthopedic surgery [23], radiation oncology [24], and general surgery [25]. However, this trend raises a concern about a lack of more inclusive participation as not many influencers were from developing countries.

The participation of healthcare providers of all nationalities in the X discourse in IBD at a global scale is highly relevant as the disease has transitioned from a few sporadic cases among people of Western European descent to a global IBD population with accelerating emergence across the globe [26]. Multiple studies have emphasized the rising IBD burden in developing nations, especially in the Asian continent, with added constraints of misdiagnosis or underdiagnosis pointing towards actual prevalence being much higher than the reported data [27,28]. A study [29] analyzing the inflammatory disease burden in Asia has observed that India, one of the developing countries, was the second-largest contributor to the IBD burden after the US. However, there were no influencers in this cohort from Asia, suggesting a huge opportunity for the professionals from this continent to provide information regarding this disease to the population of their regions.

It is crucial to understand that the ranking system for social media influence does not necessarily correlate with a physician's ability to provide care to his patients or carry out research. However, the results of the present study pointed towards a moderately positive correlation (r=+0.488) between academic productivity, reflected by the h-index, and active presence on X with regard to IBD (Figure 4). This aligns with previous studies on the top 100 influencers in cardiology [21] as well as neurosurgery [22] wherein r values of 0.32 and 0.35 were reported respectively. This implies that health professionals active in research also participate in social media engagements, which can be reassuring in terms of informed and credible IBD research dissemination to the masses. It is also possible that active social media engagement might also improve the h-index of researchers due to increased citations made possible by wider outreach. The greater research productivity can also be attributed to these professionals practicing in academic-oriented institutes rather than private clinics, and an earlier study [18] analyzing the X accounts of top gastroenterologists has revealed that 78.1% of gastroenterology social influencers worked in academia, and received funding for research, which translated into research productivity with an average h-index of 21.6.

Our study also noted a higher h-index among male influencers compared to their female counterparts. This is in line with the findings of previous studies [20,21], which also demonstrated a higher h-index among male influencers, and this highlights the need to further improve the active participation of women at all hierarchical levels in the field of healthcare.

Despite its insightful findings, this study has certain limitations, primarily its reliance on the algorithm by Cronycle. The use of another algorithm to identify IBD influencers might have resulted in some variations in our findings. Furthermore, the dynamic nature of the presented data in the constantly evolving digital age is another dilemma, as the order of ranks could be influenced by the posts the influencer made after the collection of our data. The non-acquaintance or non-engagement of healthcare experts in the field of IBD on X or usage with other overlapping hashtags but not queried IBD keywords might have undermined their impact with regard to the present work. Despite these limitations, our study’s finding about the strong correlation between Twitter scores and the h-index is significant.

## Conclusions

Social media plays a pivotal role in the dissemination of information about IBD. Our study findings highlight the prominent participation of gastroenterologists and IBD advocates on X in contributing toward the better management of IBD. The majority of the influential voices were from developed nations with male professionals exhibiting both higher social and academic influence. Hence, there is a need for a more equitable involvement in terms of gender and geographical location for the sharing of quality and relevant information, thereby paving the way for the efficient management of this globally prevalent condition with a rapidly evolving epidemiology.
